# Interaction of Microplastics with Emerging Organic Pollutants: A Study on Atrazine Adsorption and Phytotoxicity

**DOI:** 10.3390/toxics13040257

**Published:** 2025-03-29

**Authors:** Luan Gabriel Xavier de Souza, Francisco Javier Cuba Teran, Renata Medici Frayne Cuba, Andréa Rodrigues Chaves, Kellen Cristina da Silva

**Affiliations:** 1 Civil and Environmental School, Federal University of Goiás, Goiania 74605-220, Brazil; luan.gabriel@ufg.br (L.G.X.d.S.); renatafrayne@ufg.br (R.M.F.C.); kellen_cristina@discente.ufg.br (K.C.d.S.); 2 Chemistry Institute, Federal University of Goiás, Goiania 74001-970, Brazil; andrea_chaves@ufg.br

**Keywords:** microplastic, phytotoxicity, atrazine

## Abstract

The adsorption of atrazine (ATZ) onto pristine and aged polyethylene microplastics (MPs) was investigated in distilled water (DW) and hydroponic nutrient-enriched water (EW) to evaluate its phytotoxic effects on *Lactuca sativa* germination. Aged microplastics (AMPs) exhibited higher ATZ adsorption in both conditions: 0.646 mg/g (14.49%) in DW and 0.742 mg/g (15.87%) in EW, compared to 0.405 mg/g (9.08%) and 0.504 mg/g (10.78%) for pristine microplastics (PMPs), respectively. This increase was attributed to photodegradation-induced surface modifications on MP, including increased roughness and the formation of oxygenated functional groups. The phytotoxicity assays showed that ATZ adsorbed onto AMPs inhibited seed germination more severely, with a maximum inhibition of 34% at 2 mg/L, evidencing that microplastic aging enhances ATZ adsorption and increases toxicity risks in aquatic environments, particularly under eutrophic conditions. The combined presence of MP and ATZ resulted in greater toxicity, attributed to a synergistic effect, as observed in dry and wet mass inhibition. These findings indicate that pollutant interactions amplify negative impacts on plant development. Furthermore, ATZ primarily affects root growth through direct physical contact with MP rather than via desorption into water.

## 1. Introduction

Plastic pollution is an escalating global issue affecting various environmental compartments, including soil, water, and air [1]. The global production of approximately 430 million tons of plastic annually raises serious concerns [2]. A significant concern is the fragmentation of plastics into MPs, a process occurring both naturally, through macroplastic degradation, and intentionally via industrial production for applications in construction, cosmetics, and nautical industries [3].

MP particles, typically ranging from 1 to 5 mm in size, have been extensively studied in the context of plastic pollution due to their adverse effects. These effects range from developmental modifications in photosynthetic organisms—leading to oxidative stress, as observed in phytotoxicity tests [4]—to impacts on top-tier organisms, particularly marine species, where MP ingestion frequently results in gastrointestinal obstruction and suffocation [5].

Beyond their direct effects, MPs can modify pollutant toxicity and bioavailability [6,7]. In aquatic environments, these particles interact with organic and inorganic pollutants, promoting contaminant retention through adsorption processes [8]. Under specific environmental conditions like weathering, salinity, pH, and heavy metals, these pollutants may transfer onto MPs and later be released through desorption mechanisms [9,10,11]. Studies have demonstrated that MPs can adsorb pesticides, including ATZ, affecting their environmental fate and potential bioavailability [12,13]. Research indicates that MPs enhance the persistence of ATZ in aquatic environments, increasing its potential for bioaccumulation and ecological risks [14]. Furthermore, microbial aging and environmental factors alter MP’s surface characteristics, modifying ATZ adsorption behaviors [15].

Investigating these interactions is crucial due to their potential to exacerbate environmental pollution, particularly through interactions with widely used organic contaminants such as pesticides. With global population growth, the demand for agricultural pesticides increases proportionally to ensure food supply [16]. The widespread use of these substances in agriculture raises concerns regarding their environmental impacts [17], particularly their growing presence in soils [18] and aquatic compartments [19]. Among the numerous pesticides, some are of particular concern due to their extensive use, prolonged persistence, long residual time, and stability—such as ATZ [20].

ATZ is extensively used and is among the most consumed pesticides in Brazil [21] and globally. AZT is applied as a pre- and post-emergent herbicide in agricultural practices, particularly in maize, sugarcane, and sorghum cultivation [22]. Classified as a highly hazardous pesticide by the Pesticide Action Network (PAN), ATZ contamination in natural environments, including irrigation water and agricultural drainage [23,24], represents a significant concern. Due to its moderate to high persistence in soil and aquatic environments, ATZ can remain in ecosystems for weeks to months, particularly in groundwater, where degradation is slower, leading to long-term exposure risks [25]. Moreover, it has been associated with adverse health effects, including potential endocrine disruption and reproductive toxicity [26,27]. The interaction between MPs and ATZ is an emerging environmental issue, as such interactions are becoming increasingly frequent, potentially leading to combined contamination [28].

Previous studies [29,30,31,32] have primarily focused on MP–ATZ interactions in single-phase systems. However, the influence of environmentally relevant water compositions, particularly under nutrient-rich conditions, remains underexplored. Additionally, while many studies have examined MP and pesticide interactions from a physicochemical perspective, the combined effects of these contaminants on plant health and phytotoxicity have received limited attention. By assessing MP–ATZ interactions in both DW and EW and incorporating phytotoxicity evaluations, our study provides a more comprehensive understanding of how these pollutants behave in real aquatic environments and their potential ecological risks.

The effects of ATZ on the environment extend beyond target organisms, disrupting the life cycle of various species due to water and soil contamination [31]. ATZ undergoes chemical transformations that enhance its conversion into toxic and persistent compounds [32]. Its toxicity has been reported in humans (disrupting hypothalamic control and affecting primary hepatocytes and hepatic stem cells), as well as in non-target plants and microbes (inducing oxidative stress and reducing population size) [33].

Thus, the objective of this research is to investigate, through adsorption and desorption experiments, the interaction between ATZ and MPs. This study aims to evaluate this interaction in different matrices, including DW and hydroponic EW. Additionally, the phytotoxic effects of this interaction on *Lactuca sativa* will be assessed through phytotoxicity assays.

## 2. Materials and Methods

### 2.1. Simulation of Environmental Conditions

This study was conducted under simulated environmental conditions, using DW (pH 6) and hydroponic EW (pH 7), formulated with salts and minerals commonly found in hydroponic systems. These matrices were employed to explore the interactions between MPs and contaminants in different representative scenarios to evaluate the influence of water composition on the interactions between PMPs, AMPs, and ATZ.

### 2.2. Microplastic Acquisition and Photodegradation

Polyethylene microspheres, with an average diameter of 0.5 mm, were purchased from Bianquimica, São Paulo, Brazil. The samples underwent photodegradation in an accelerated aging chamber for 120 days. Continuous UV radiation was applied, maintaining a controlled average temperature of 32 °C. To ensure uniform exposure to radiation, weekly redistribution of the microspheres within the chamber was performed. These conditions were selected based on their relevance in simulating environmental aging processes and assessing the photodegradation behavior of contaminants. The use of an accelerated aging chamber with continuous UV radiation and a controlled temperature of 32 °C aligns with established methodologies for evaluating the stability and transformation of pollutants under realistic yet controlled conditions [34,35].

Previous studies have demonstrated that the fragmentation of micro- and nanoplastics, induced by photo-oxidation in accelerated aging chambers, varies among different polymers, highlighting the importance of simulating environmental conditions to understand these processes [35]. The 120-day exposure period was chosen to capture potential degradation pathways over extended exposure, simulating long-term environmental interactions. These parameters maintain consistency with previous studies, facilitating comparison and validation of the observed results.

### 2.3. Microplastic Characterization

MPs were characterized based on their morphological and physicochemical properties. Morphological analysis was conducted using scanning electron microscopy (SEM) (Jeol, JSM-6610 Tokyo, Japan), while the physicochemical characterization included zeta potential (ZP) measurements (Zetasizer Nano Series ZS, Marvel, Worcestershire, UK) and Fourier transform infrared spectroscopy (FTIR) to assess the surface modifications in MPs (Bruker Vertex 70, Ettingen, Germany). The FTIR measurements were conducted with a resolution of 4 cm⁻^1^, using 32 scans and an acquisition range of 4000–400 cm⁻^1^. Prior to analysis, the samples were pre-filtered using a 0.4 µm syringe filter (PTFE MerckMillipore, Darmstadt Hesse, Germany) to remove impurities and undesirable particles. The filters were pre-saturated before use, and the initial filtrate was discarded.

The Carbonyl Index (CI) was calculated to evaluate MP aging, determined as the ratio of the carbonyl absorption band area (1850–1650 cm⁻^1^) to the reference methylene band area (1500–1420 cm⁻^1^), as shown in Equation (1).

(1) $$CI=\frac{Carbonyl\;band\;area\;(1850-1650\;cm^{-1})}{Reference\;band\;area\;(1500-1420\;cm^{-1})}\;$$

### 2.4. Adsorption and Desorption Assays

Preliminary assays were conducted to determine the optimal concentration of polyethylene microspheres based on their adsorption capacity. A 2 mg/L ATZ solution was used, varying the MP mass from 5 mg to 30 mg in 50 mL of ATZ solution. The samples were continuously agitated (120 rpm) for 48 h. The removal efficiencies and adsorption capacities were calculated using Equation (2).

(2) $$q_{t}=\frac{(C_{0}-C_{t})V}{m}\;$$

where *q_t_* is the adsorption capacity, *C*_0_ and *C_t_* represent the initial and final concentrations of ATZ, *V* is the solution volume, and *m* is the mass of microplastic.

To determine the adsorption kinetics, the effect of the contact time was evaluated at intervals of 0, 15, 30, 60, 240, 1440, and 2880 min, enabling data modeling using pseudo-first-order (PFO) and pseudo-second-order (PSO) models. The experiments were conducted using 0.025 g of MPs (a dosage considered optimal based on preliminary experiments) in 50 mL of a 2 mg/L ATZ solution, maintained under constant agitation (120 rpm) until adsorption equilibrium was reached.

Following the determination of the optimal MP dosage in the preliminary test, adsorption isotherms were obtained by varying the initial ATZ concentration at 0.05, 0.1, 0.5, 1.0, 2.0, and 4.0 mg/L while keeping the MP mass constant. The experiments were conducted at 120 rpm for 48 h to ensure the stabilization of interactions between the contaminant and the adsorbent. The obtained data were fitted to the Langmuir and Freundlich models to determine the maximum adsorption capacity (Langmuir) and adsorption intensity (Freundlich).

For the desorption experiments, MPs that reached adsorption equilibrium under conditions of 2 mg/L ATZ and 0.025 g of MP were filtered and transferred to clean solutions, where they remained for 48 h under the same experimental conditions (120 rpm, 24 °C). The amount of released ATZ was monitored to assess the reversibility of adsorption and understand its environmental implications.

### 2.5. Phytotoxicity Assays

Phytotoxicity assays were conducted using *Lactuca sativa* seeds to evaluate the impact of MPs and ATZ on germination and root growth processes. Four treatment types were tested as follows: a negative control consisting of DW and EW without MPs or ATZ; a positive control with 0.05 mol/L of zinc sulfate (ZnSO₄) solution [36,37,38], used as a reference for known toxicity to validate the test sensitivity; treatments with isolated MPs, where seeds were exposed to MPs in distilled or enriched water without ATZ; and treatments with MPs containing adsorbed ATZ (MP+ATZ), in which seeds were exposed to MPs pre-treated with ATZ in both aquatic matrices.

The seeds were placed in Petri dishes containing filter paper moistened with 4 mL of the corresponding solution for each treatment. The plates were incubated at a controlled temperature of 24.5 °C for 120 h in the dark. The number of MPs in each treatment was determined based on the highest ATZ adsorption capacity previously observed (0.025 g) and was conducted in triplicate. For the treatment with ATZ alone, the concentration from the adsorption assays (2 mg/L) was used. After incubation, the treatment effects were evaluated using quantitative indicators, including the Root Growth Index (ICR), Germination Index (IG), Normalized Root Growth Percentage Index (IER), and Normalized Residual Germination Percentage Index (IGN) [39].

Additionally, the inhibition percentages of the fresh and dry biomasses were assessed [4] using the obtained data. The indices were calculated using Equations (3)–(7).

Root Growth Index (ICR):

(3) $$ICR=\frac{CRA}{CRC}\;\;$$

Germination Index (IG):

(4) $$IG=ICR\times \left(\frac{SGA}{SGC}\right)\times 100\;\;$$

Normalized Root Growth Percentage Index (IER):

(5) $$IER(\%)=\frac{CMRCA-CMRCN}{CMRCN}\;$$

Normalized Residual Germination Percentage Index (IGN):

(6) $$IGN(\%)=\frac{GERM\;sample-GERM\;control}{GERM\;control}\;\;$$

Inhibition of Dry and Fresh Biomass (INM) (%):

(7) $$\frac{Mass\;in\;Control-Mass\;in\;Treatment}{\left(Mass\;in\;Control\right)}\times 100\;$$

Variable Definitions:

ICR: Root Growth Index.

CRA: Root Growth in Sample.

CRC: Root Growth in the Control.

IG: Germination Index.

SGA: Number of Germinated Seeds in Sample.

SGC: Number of Germinated Seeds in Negative Control.

IER: Normalized Root Growth Percentage Index

CMRCA: Average Radicle Length in Each Sample

CMRCN: Average Radicle Length of Germinated Seeds in the Control.

IGN: Normalized Residual Germination Percentage Index.

GERMsample: Average Percentage of Germinated Seeds in Sample.

GERMcontrol: Percentage of Germinated Seeds in Negative Control.

These calculations followed the methodologies described in previous studies [40]. The NRGPI and NRGPI values were used to classify the toxicity levels according to Table 1.

All treatments were conducted in triplicate, and the error bars presented in the graphs represent the standard deviation of the means (mean ± SD). Standard deviation values were calculated using the statistical software packages RStudio (version 2024,12,1-563) and Microsoft Excel (Microsoft Office Home and Business 2016). Prior to statistical analyses, the Shapiro–Wilk normality test (α = 0.05) was performed to assess the normality of the residuals. When normality was confirmed, and ANOVA indicated significance (*p* ≤ 0.05), Tukey’s multiple comparisons test was applied to identify significant differences between treatments. For data that did not meet the normality criteria, the non-parametric Kruskal–Wallis test was used, followed by Dunn’s multiple comparisons test.

## 3. Results

### 3.1. Surface Analysis of Microplastic by SEM and FTIR

**Figure 1:**
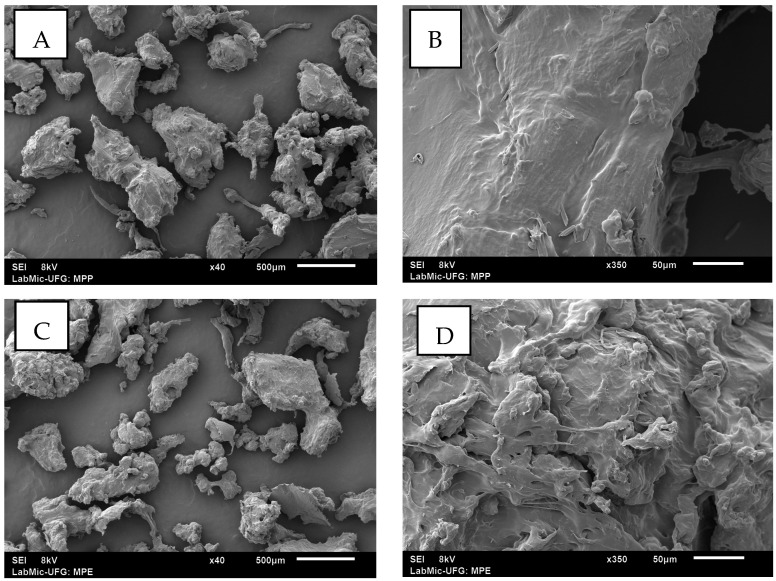
Image (**A**): SEM of PMPs (40×), displaying irregular morphology and rough edges, scale bar: 500 µm. Image (**B**): SEM of PMPs (350×), scale bar: 50 µm, exhibiting a smooth surface with no deformations. Image (**C**): SEM of AMPs (40×), scale bar: 500 µm, with fragmented particles and irregular distribution (scale bar: 500 µm). Image (**D**): AMPs (350×), showing a worn texture and thin layers (scale bar: 50 µm), scale bar: 50 µm.

SEM revealed significant structural changes in AMPs (Figure 1A,B). While the PMPs exhibited smooth and homogeneous surfaces (Figure 1C,D), the AMPs displayed roughness, cracks, and surface cavities, characteristics indicative of structural degradation.

**Figure 2:**
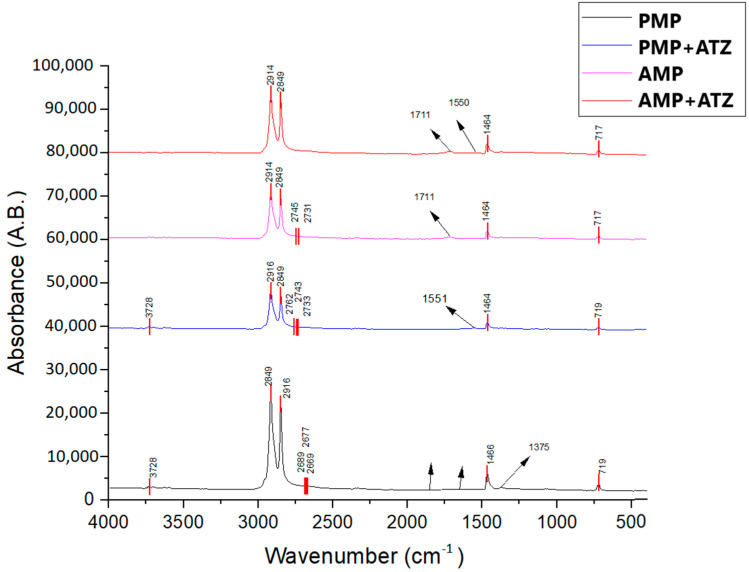
FTIR spectra of PE plastic fragments: The AMPs are shown in red, and the control PMPs are shown in black.

FTIR was employed to assess the changes in the functional groups of MP particles before and after ATZ adsorption, as well as the structural modifications in the MPs due to aging (Figure 2). The spectral peaks observed for the PE MPs were attributed to -CH₂- stretching at 2915 cm⁻^1^ and 2848 cm⁻^1^, -CH₂- bending at 1464 cm⁻^1^, and -(CH₂)n- rocking at 719 cm⁻^1^. After adsorption, spectral peaks were identified at 3254 cm⁻^1^ (-NH stretching) and 1550 cm⁻^1^ (thiotriazine stretching), which are characteristic of ATZ but appeared with low intensity, suggesting that the interaction between this compound and both PMPs and AMPs was primarily physical [15]. Aging of polyethylene MPs due to UV radiation exposure for 120 days resulted in significant chemical changes on their surfaces, as observed through the Fourier transform infrared (FTIR) spectroscopy analyses. These alterations are primarily evident in the spectral regions corresponding to the surface oxygenated groups, such as hydroxyl groups (3600–3000 cm⁻^1^), carbonyls (1800–1400 cm⁻^1^), and esters and vinyl groups (1400–800 cm⁻^1^), as reported in previous studies [41,42].

One of the main spectral modifications observed in the FTIR spectrum was within the 1800–1500 cm⁻^1^ range, where carbonyl (C=O) peak reshaping occurred, with a broad peak at 1713 cm⁻^1^, characterizing the formation of carbonyl groups due to UV-induced oxidation [43,44]. This peak was more pronounced in AMPs compared to the PMPs, as also reported by Zidar et al. [45]. Additionally, in the 3600–3000 cm⁻^1^ region, short vibration remodeling was observed in a broad band associated with the presence of hydroxyl (-OH) groups.

To quantify these structural modifications, the Carbonyl Index (CI) was calculated as the ratio of the carbonyl absorption band area (1850–1650 cm⁻^1^) to the reference methylene band area (1500–1420 cm⁻^1^). In PMPs, the CI was determined to be 1.623, whereas in AMPs, it reached 2.357, representing a significant 45.2% increase in carbonyl group formation due to aging. The rise in the CI further supports the chemical degradation of polymer chains, confirming significant alterations in the material’s surface properties upon aging [46].

These results demonstrate that aging not only promoted the formation of carbonyl groups but also introduced other oxygenated functional groups, such as hydroxyls and carbonyls, increasing surface polarity. This photodegradation process altered both the chemical and physical properties of the MPs, making them more reactive and potentially more susceptible to interactions with polar environmental compounds such as ATZ.

**Figure 3:**
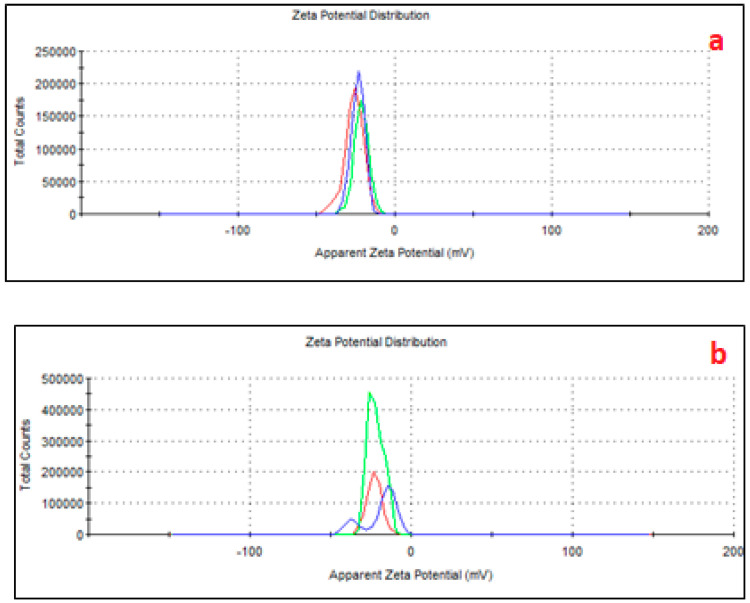
Zeta potential of AMPs (**a**) and PMPs (**b**), with adsorbed ATZ. The line colors refer to three replicates of the same sample.

The zeta potential measurements (Figure 3) further confirmed these changes, showing more negative values in AMPs (−32 mV) compared to PMPs (−26 mV), reinforcing the higher capacity of AMPs to interact with polar molecules [18,20]. These structural and chemical modifications highlight the impact of aging on the reactivity and environmental behavior of MPs [47], showing a greater surface density of functional groups, such as carbonyl and hydroxyl, which are introduced during the aging process due to oxidative weathering. In contrast, PMPs, with a less negative zeta potential, possessed a relatively lower surface reactivity, limiting its ability to engage in similar interactions.

Even considering the negatively charged microplastic, ATZ is a neutral to weakly basic herbicide with a pKa of 1.68, meaning it remains predominantly neutral at environmental pH levels (e.g., pH 5–8). However, its adsorption onto aged MPs can modify the surface properties by introducing hydrophobic patches or altering the charge distribution, thereby reducing overall surface negativity or even creating localized positive sites. Consequently, MPs with adsorbed ATZ can engage in electrostatic interactions with plant roots, with the nature of this interaction depending on the surface charge modifications induced by ATZ adsorption, as we see in Figure 3.

### 3.2. Adsorption Capacity

The analysis of ATZ adsorption on PMPs and AMPs, considering the influence of the surrounding medium, reveals important insights into the adsorption mechanisms and the factors that influence them.

The obtained data indicate that both the properties of the MPs and the characteristics of the medium play significant roles in sorption efficiency. These variables should be evaluated together for a comprehensive understanding of contaminant behavior in aquatic environments. In the adsorption capacity assay, the results indicated that adsorption was most efficient with 25 mg of microplastics. This mass was selected due to its higher *q*e value compared to the other tested quantities.

**Figure 4:**
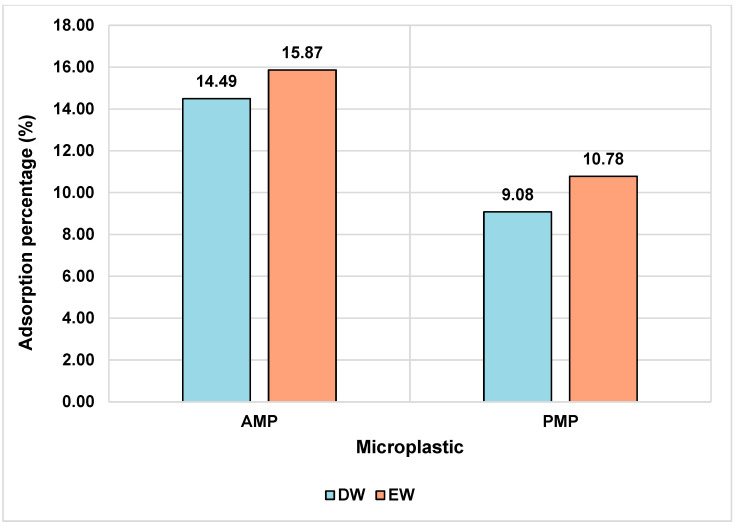
Sorption efficiency at equilibrium for ATZ and PMPs in DW and EW media. Experimental conditions: temperature = 25 °C; ATZ concentration = 2.0 mg L⁻^1^; contact time = 24 h.

When comparing AMPs with PMPs, a slight difference in ATZ adsorption capacity is observed. In EW, the AMPs adsorbed a slightly higher amount of ATZ (0.742 mg/g) compared to the PMPs (0.5042 mg/g), as clearly depicted in Figure 4. This behavior may be attributed to the chemical modifications occurring on the MP’s surface during the aging process. The FTIR analysis suggested the emergence of carboxyl (C=O) groups due to the oxidation of PE particles. These functional groups may interact with the ethylamine moiety of ATZ molecules, which acts as a hydrogen bond donor [48].

Although the data indicate a slight advantage for AMPs in terms of ATZ sorption efficiency, the observed differences are relatively small. This suggests that the affinity of ATZ for both types of MPs in DW and EW media is low. Despite the modest absolute amount of ATZ adsorbed by the MPs (0.3 mg/L), it significantly exceeds the maximum allowable limits in drinking water, such as the 0.1 μg/L threshold established by the European Union [49].

As shown in Figure 4, MPs in the enriched medium exhibited higher adsorption values for AMPs and PMPs. This phenomenon may be related to the presence of ions, which influence the adsorption process through mechanisms such as electrostatic shielding, salting-out effects, and extrusion. The presence of NaCl, for example, tends to induce a salting-out effect on the adsorption of hydrophobic chemical compounds [50], which may have influenced the results observed in this study, given the presence of this salt in the enriched solutions.

Furthermore, the presence of certain inorganic ions, beyond sodium and chloride, in natural water bodies can also influence adsorption. Studies have demonstrated that ions such as Cl⁻, SO₄^2^⁻, and HCO₃⁻ enhance ATZ adsorption capacity to varying degrees, while ions such as Mg^2^⁺ and Ca^2^⁺ may inhibit this process. For instance, in the presence of Mg^2^⁺, the adsorption capacity of PMPs decreased by 25.9%, whereas AMPs exhibited only a 4% reduction. This suggests that electrostatic attraction has a limited effect [18].

This phenomenon occurs mainly because ions such as Mg^2^⁺ and Ca^2^⁺ compete for adsorption sites on MPs. Being positively charged, they can be adsorbed onto the particles via electrostatic attraction, occupying the same adsorption sites as ATZ [51].

### 3.3. Adsorption Kinetics

**Figure 5:**
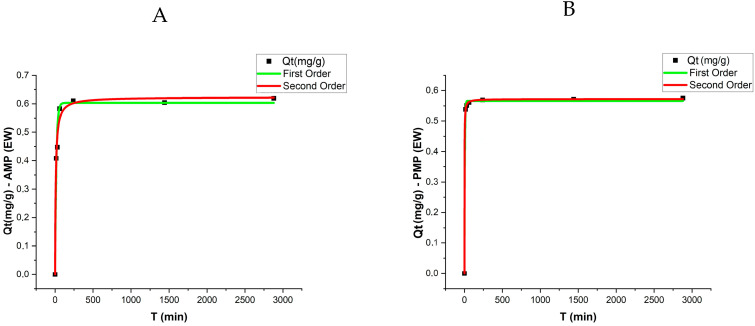

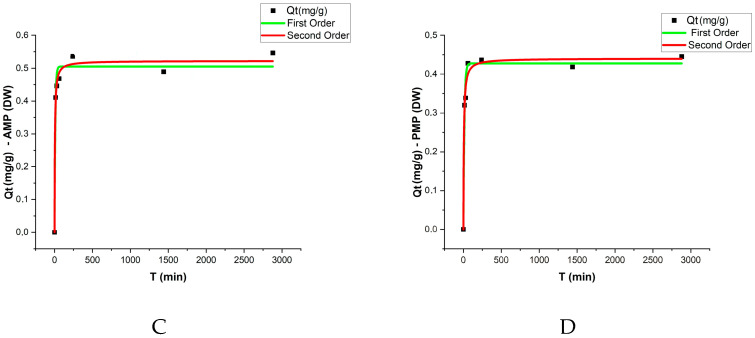
Sorption kinetics of ATZ on (**A**) AMPs in EW, (**B**) PMPs in EW, (**C**) AMPs in DW, and (**D**) PMPs in DW. Experimental conditions: temperature = 25 °C; polyethylene concentration = 0.5 g L⁻^1^.

The adsorption kinetic results were evaluated using pseudo-first-order and pseudo-second-order models, with the respective parameters presented in Table 2 and Figure 5. The analysis revealed that equilibrium was reached after approximately 48 h, with a sharp increase in adsorption rates during the initial stages of the experiment. The qe values (adsorption capacity at equilibrium) varied among treatments, with AMPs exhibiting slightly higher adsorption capacities compared to PMPs in both matrices (DW and EW).

For the pseudo-first-order model, the qe values for PMPs were 0.427 mg/g in DW and 0.566 mg/g in EW. In contrast, for AMPs, these values increased to 0.505 mg/g and 0.603 mg/g, respectively. The determination coefficients (R^2^) for this model ranged from 0.968 to 0.9985, indicating a good fit of the experimental data to the model under both conditions.

In the pseudo-second-order model, the qe values were slightly higher, reaching 0.522 mg/g for AMPs in DW and 0.623 mg/g in EW. These values suggest that the adsorption mechanism is influenced by stronger chemical interactions between ATZ and AMPs. The k₂ coefficients for both PMPs and AMPs were higher in EW, with PMPs showing a particularly high k₂ value (1.75) and an excellent fit (R^2^ = 0.9998).

When comparing the two models, the pseudo-second-order model exhibited higher R^2^ coefficients (ranging from 0.9833 to 0.9998), suggesting that this model better describes the adsorption process of ATZ onto MPs, particularly in EW.

Figure 5 presents the experimental data along with the model fittings. These results highlight the influence of aging and the enriched aqueous matrix on the adsorption kinetics, demonstrating that the higher polarity of AMPs enhance ATZ retention.

### 3.4. Isotherms

**Figure 6:**
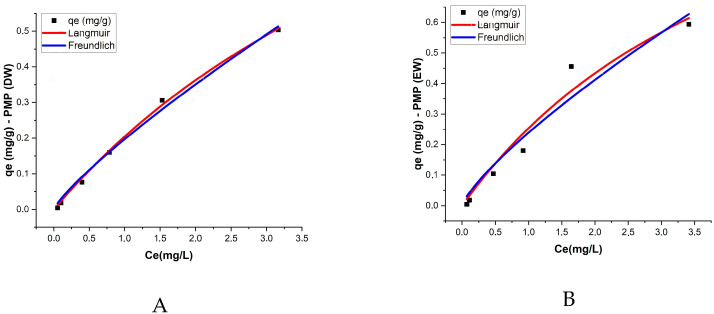

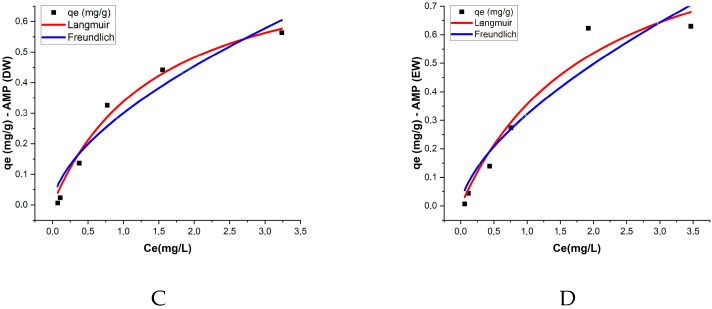
Sorption isotherms for (**A**) AMPs in EW, (**B**) AMPs in EW, (**C**) AMPs in DW, and (**D**) PMPs in DW. Experimental Conditions: temperature = 25 °C; polyethylene concentration = 0.5 g/L.

The Langmuir and Freundlich isotherm models were applied to describe the adsorption of ATZ onto both PMPs and AMPs in two aquatic matrices: DW and EW. The Langmuir model provided the best fit for most treatments, with the R^2^ values exceeding 0.9, indicating good agreement with the experimental data. This result suggests that adsorption occurs in monolayers with a homogeneous adsorption surface (Table 3, Figure 6).

In PMPs in DW, the maximum adsorption capacity (qm) was 1.612 mg/g, while the Langmuir constant (KL) was 0.145 L/mg, reflecting a moderate affinity between ATZ and the PMP surfaces. For AMPs in DW, the maximum adsorption capacity decreased to 0.841 mg/g, but the K_L_ constant significantly increased to 0.671 L/mg, indicating that despite the reduction in total adsorption capacity, the affinity between AMPs and ATZ was higher. In EW, PMPs exhibited a qm of 1.493 mg/g and a KL of 0.204 L/mg, while AMPs had a lower qm of 1.076 mg/g but a higher KL of 0.495 L/mg.

Wang et al. [52] reported qe values for ATZ adsorption onto different types of MPs that are of the same order of magnitude as those obtained in the present study, specifically 1.349 mg/g, 1.992 mg/g, and 1.148 mg/g. After aging, the authors observed increases of 84%, 78%, and 95% for polystyrene, polyethylene, and polypropylene, respectively. In contrast, our study reported a decrease in qm by 52% in DW and 74% in EW after aging. Prolonged UV exposure, as applied in our study, may lead to more extensive polymer degradation, fragmentation, and morphological alterations, such as reduced surface area or pore collapse, which could negatively impact adsorption capacity. When comparing the qm and K_L_ values obtained by Wang et al. [52] with those in our study, the increase in K_L_ values for AMPs in both cases suggests that differences in adsorption behavior may be attributed to distinct morphological changes resulting from variations in the aging duration. In the present study, MPs were exposed to UV radiation for 120 days, whereas Wang et al. [52] applied UV exposure for only 96 h. According to Chen et al. [53], prolonged UV exposure induces more pronounced alterations in the surface structure of MPs. This is further supported by Zhang et al. [54], who reported a reduction in the surface area of three types of MPs after 30 days of UV aging. The Freundlich model exhibited a slightly lower R^2^ compared to the Langmuir model, ranging from 0.901 to 0.991, yet still demonstrated good agreement with the experimental data, suggesting that the process occurs on heterogeneous surfaces with non-uniform energetic interactions. The values of the K_F_ constant, which reflects the adsorption capacity, were higher for AMPs in both matrices, with K_F_ = 0.3 in DW and K_F_ = 0.321 in EW (Table 3; Figure 6).

These results indicate that the aging of MPs promotes additional interactions [55]. The presence of ions in enriched water intensified ATZ retention, possibly due to the stabilization of chemical interactions between the contaminant and oxygenated functional groups in AMPs.

Isothermal analysis using the Langmuir and Freundlich models indicated that both models were applicable to the ATZ adsorption process (R^2^ > 0.95), suggesting that the interaction between PMPs and AMPs and ATZ in the aquatic environment is not limited to monolayer adsorption but also includes chemical adsorption mechanisms. For AMPs, the maximum adsorption capacity (Qₘ) was significantly higher than in PMPs, demonstrating that aging alters the surface properties of MPs, increasing their affinity for ATZ.

The binding between chemical compounds and polymers is often associated with adsorption and distribution processes [56]. While fully crystalline polymers allow only surface adsorption, semicrystalline polymers, such as polyethylene MPs, enable both surface adsorption and penetration into their amorphous internal regions [57]. As incomplete crystalline polymers, MPs allow ATZ to be adsorbed on the surface and distributed internally. However, due to the slightly alkaline nature of ATZ [18], electrostatic interactions exert limited influence on the adsorption process.

### 3.5. Desorption and Phytotoxicity

During the desorption stage, ATZ molecules were not detected in the desorbed water from MPs with ATZ, indicating that the remaining fraction of ATZ remained strongly adsorbed onto the MPs’ surfaces, particularly in AMPs, and was not released under the experimental conditions. These findings suggest that ATZ sorbed onto MPs was resistant to the desorption processes applied. Consequently, phytotoxicity tests were conducted in the presence of MP particles with adsorbed ATZ.

#### 3.5.1. Germination

**Figure 7:**
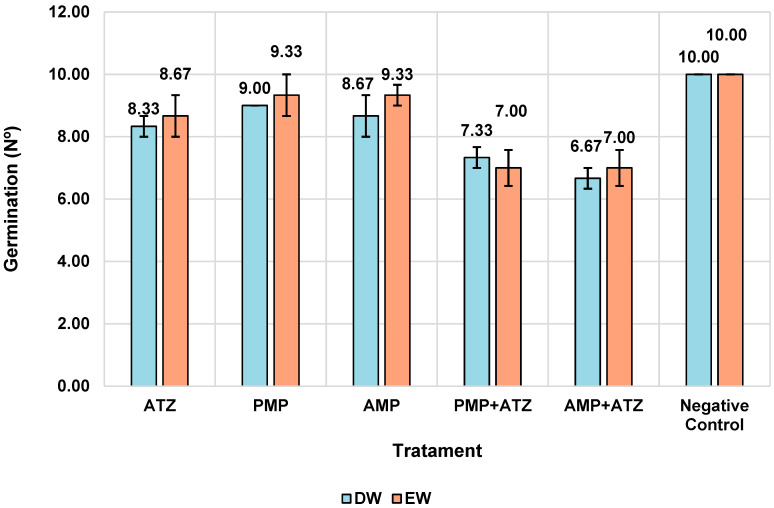
Mean germination numbers with confidence intervals by treatment and medium.

The germination assays demonstrated significant reductions in the number of germinated *Lactuca sativa* seeds in response to treatments with MPs and ATZ. Treatments combining AMPa and ATZ exhibited the highest reductions, with 34% in DW and 30% in EW. Treatments with PMPs and ATZ showed lower reductions, indicating a less severe impact compared to AMPs. The negative control and treatments with PMPs alone (without ATZ) exhibited germination rates close to the reference values, indicating a lower phytotoxic impact. Figure 7 illustrates the distribution of the mean germination values across different treatments and aquatic matrices.

#### 3.5.2. Dry Mass

**Figure 8:**
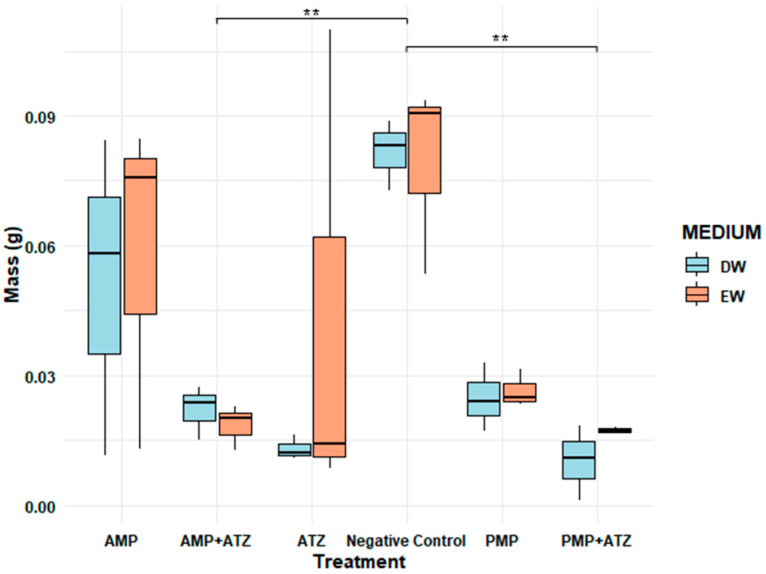
A comparison of *Lactuca sativa* dry mass (g) under different treatments in two aquatic matrices (DW; EW). The treatments include ATZ alone, a negative control, AMPs, AMPs with ATZ (AMP+ATZ), PMPs, and PMPs with ATZ (PMP+ATZ). The bars represent interquartile ranges, while the dots indicate extreme values. The asterisks (*) denote statistically significant differences, ** very significant.

The dry mass results for *Lactuca sativa* revealed significant variations across different treatments and aquatic matrices. The highest inhibition percentages were observed in treatments with AMPs combined with ATZ (AMP+ATZ), reaching 87.5% in DW and 78.2% in EW (Figure 8). These values were significantly higher than those of other treatments, as indicated by statistical tests (*p* < 0.05).

**Figure 9:**
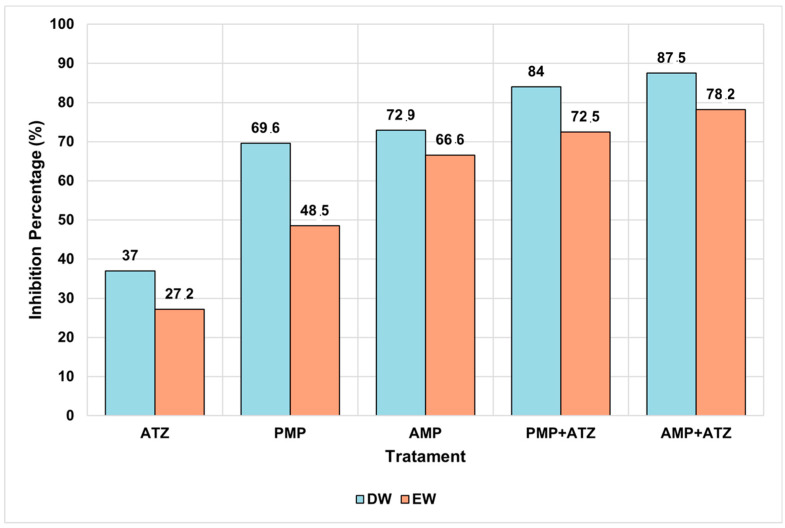
The percentage inhibition of *Lactuca sativa* dry mass under different treatments (ATZ, PMP, AMP, PMP+ATZ, and AMP+ATZ) in DW and EW. The treatments include ATZ alone, AMPs, AMPs with ATZ (AMP+ATZ), PMPs, and PMPs with ATZ (PMP+ATZ).

Treatments with PMP+ATZ also exhibited high inhibition, with 84.0% in DW and 72.5% in EW, showing statistically significant differences compared to the treatments with MP or ATZ alone. Treatments with MPs alone showed lower inhibition values: for AMPs, 72.9% in DW and 66.6% in EW, while for PMPs, the inhibition values were 69.6% in DW and 48.5% in EW (Figure 9).

Conversely, the negative control and ATZ alone presented the lowest inhibition percentages, with 37.0% in DW and 27.2% in EW, showing no statistically significant differences between them. Statistical analysis indicated that the presence of MPs, either alone or combined with ATZ, significantly increased the dry mass inhibition compared to the control, with the AMP+ATZ treatment exhibiting the greatest impact.

#### 3.5.3. Fresh Mass

The fresh mass analysis revealed consistent patterns among the different treatments. Treatments combining AMPs and ATZ exhibited the highest fresh mass inhibition percentages, reaching 58.5% in DW and 46.7% in EW. These values highlight the pronounced impact of these treatments compared to the others (Figure 9). Statistical analysis confirmed that the AMP+ATZ combination was highly significant (*p* = 0.00007) compared to the ATZ-only treatment, demonstrating the synergistic interaction between AMPs and ATZ.

**Figure 10:**
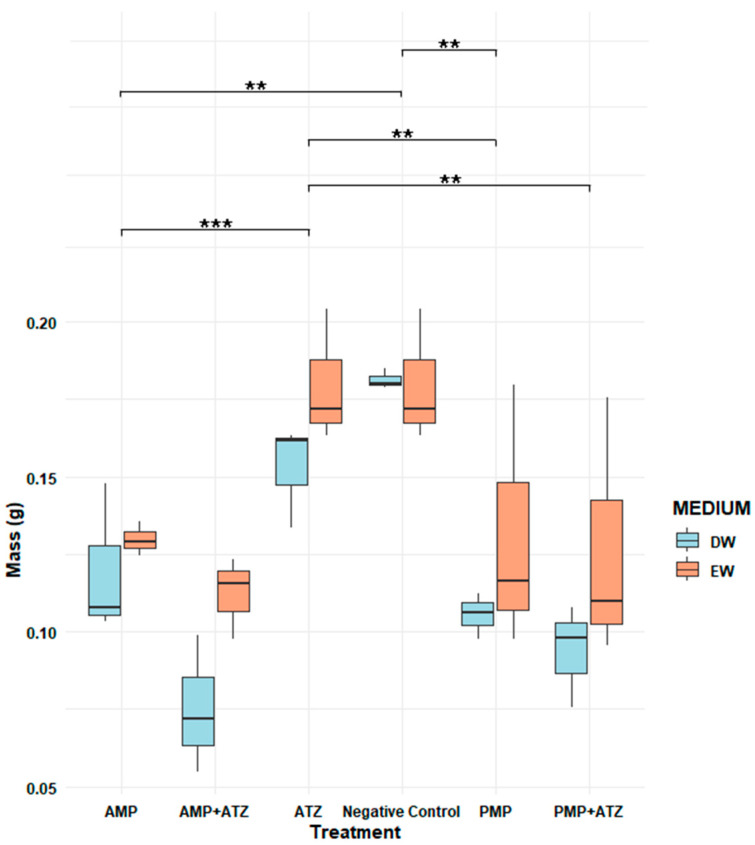
Comparison of the fresh mass (g) of Lactuca sativa under different treatments in two aquatic matrices ( DW and EW). Treatments include ATZ alone, a negative control, AMP, AMP +ATZ, PMP, and PMP+ATZ. Bars represent interquartile ranges, and dots indicate extreme values. The asterisks (*) denote statistically significant differences, ** very significant, and *** highly significant.

Treatments with PMPs combined with ATZ also showed significant inhibition, with percentages of 39.8% in DW and 38.4% in EW, though these were lower than those observed for AMP+ATZ. These results suggest that PMPs have a lower capacity to amplify the phytotoxic effects of ATZ compared to AMPs. Statistically, the effects of PMP+ATZ were highly significant (*p* = 0.00219) when compared to the ATZ-only treatment (Figure 10).

The isolated AMP and PMP treatments exhibited lower levels of fresh mass inhibition. For AMP, the values were 48.3% in DW and 37.8% in EW, with a statistically significant difference (*p* = 0.00224) compared to the negative control. For PMPs, the inhibition values were 37.2% in DW and 33.5% in EW, also showing high statistical significance (*p* = 0.0006).

**Figure 11:**
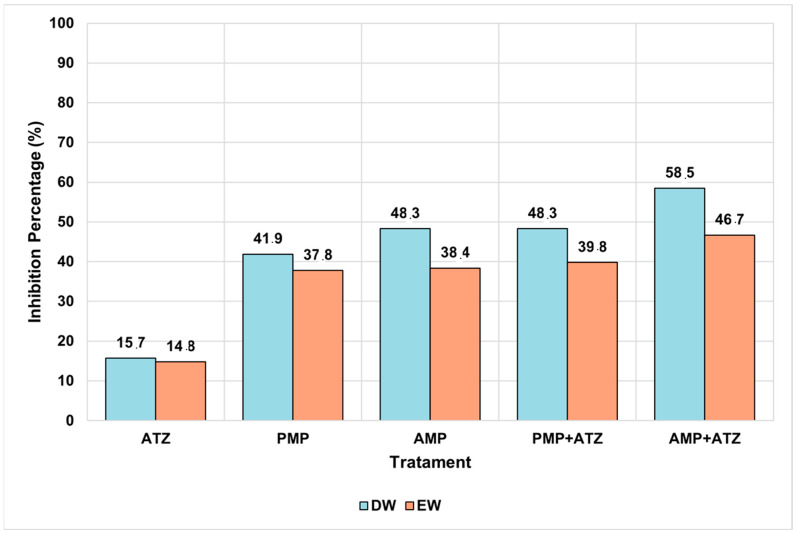
The percentage inhibition of *Lactuca sativa* fresh mass under different treatments (ATZ, PMP, AMP, PMP+ATZ, and AMP+ATZ) in DW and EW.

Overall, the combined treatments, particularly AMP+ATZ, produced the most pronounced phytotoxic effects, with statistically significant differences compared to the negative controls and isolated treatments. Additionally, adjusted *p*-values reinforced the robustness of the findings, highlighting the role of AMS in amplifying the toxic effects of ATZ (Figure 11).

The results (Figure 7, Figure 8, Figure 9, Figure 10 and Figure 11) show that ATZ alone exhibited relatively low inhibition of lettuce root growth, whereas ATZ adsorbed onto AMPs significantly intensified the phytotoxic effects. The phytotoxicity assays demonstrated that root elongation and biomass were more severely affected in the AMP+ATZ treatment compared to ATZ alone, suggesting that the presence of AMPs in the liquid medium enhances the bioavailability and toxicity of ATZ.

#### 3.5.4. SEM Analysis of Radicles and Toxicological Classification

Scanning electron microscopy (SEM) analysis and experimental data revealed that, despite the desorption assays failing to detect significant ATZ concentrations, the phytotoxicity assays demonstrated notable differences between treatments with and without the herbicide. This difference was evident even in the absence of detectable ATZ levels in DW and EW, suggesting that while ATZ was adsorbed onto MPs in small amounts, it formed strong bonds and remained retained. However, its release appears to occur only through direct contact with plant radicles.

**Figure 12:**
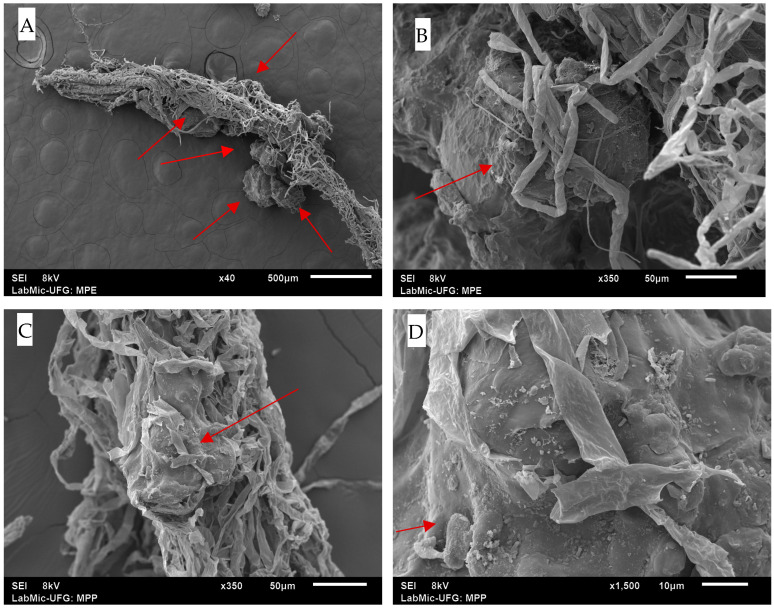
SEM images showing the interaction of MPs with *Lactuca sativa* roots: (**A**) Overview of AMPs adhering to the root surface at 40× magnification (scale bar = 500 µm); (**B**) close-up of radicle entanglement with AMPs, showing adhesion to root structures at 350× magnification (scale bar = 50 µm); (**C**) PMPs interacting with the root surface, showing dispersed and adhered fibers at 350× magnification (scale bar = 50 µm); (**D**) accumulation of PMPs on the root surface, indicating a higher adhesion density at 1500× magnification (scale bar = 10 µm). The red arrows indicate the surface of the microplastics or microplastics involved in the radicles.

The images presented in Figure 12 illustrate the interaction between *Lactuca sativa* radicles and MPs, revealing the adhesion of MPs to root surfaces. These images show the distribution of MPs predominantly at the radicle tips, supporting the hypothesis that direct physical contact was the crucial factor for localized ATZ release.

Even at residual concentrations, ATZ adsorbed onto MPs was sufficient to cause adverse effects in *Lactuca sativa*. Significant reductions in the germination rates and root length were observed, indicating that small amounts of ATZ retained by AMPs can be biologically relevant, as presented in Table 4. Treatments combining AMPs and ATZ in DW (AMP+ATZ+DW) exhibited the highest inhibition rates, with reductions of 58.52% in fresh mass and 87.50% in dry mass. In enriched water (AMP+ATZ -+ EW), the inhibition values were 46.73% for fresh mass and 78.19% for dry mass (Figure 10 and Figure 11).

**Figure 13:**
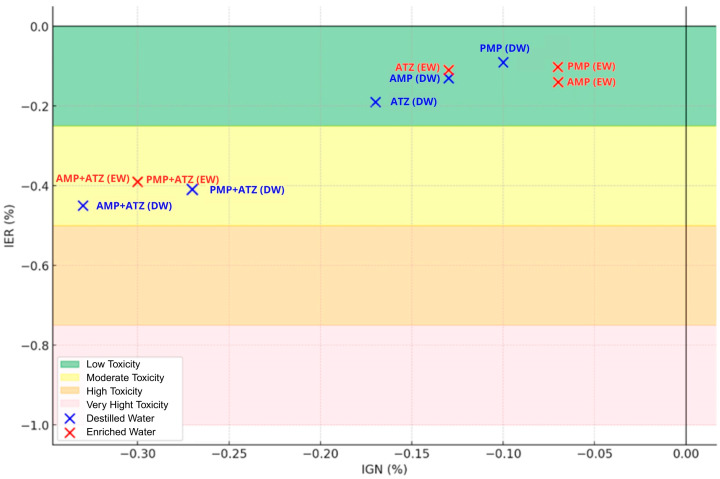
Toxicity zones: analysis by treatment and medium.

The interaction between MPs with adsorbed ATZ and plant roots can be attributed to multiple physicochemical and biological mechanisms. The adhesion of MPs to root surfaces is likely governed by hydrophobic interactions, root exudate binding, biofilm formation, electrostatic effects, and water-mediated transport. Atrazine is a hydrophobic herbicide (log Kow = 2.61), and when adsorbed onto AMPs, it may alter their surface properties, increasing their hydrophobicity and potentially enhancing root adhesion [58]. Since plant root surfaces contain hydrophobic domains, particularly due to cutin, suberin, and lignin in the cell walls [59], it is possible that hydrophobic attraction played a role in the retention of ATZ-loaded MPs on root surfaces. Figure 13 shows the toxicity zones by treatment and medium

Additionally, root exudates, which include proteins, polysaccharides, and phenolic compounds, can mediate MP–root interactions by acting as binding agents [60]. Phenolic exudates may form π-π interactions with ATZ molecules, further enhancing MP attachment to the root surface. Furthermore, mucilage, a gel-like polysaccharide secreted by roots, has been shown to trap particulate matter, including MPs, increasing their retention near the rhizosphere [61].

Another relevant factor is biofilm formation, which can influence MP–root interactions. Rhizosphere microorganisms readily colonize MP surfaces, forming biofilms that enhance adhesion [62]. These biofilms create a sticky extracellular matrix, which may further promote MP retention within root hairs or porous regions of the root surface.

From a size-dependent perspective, MPs in the nanometer to low-micrometer range may enter roots through passive apoplastic pathways (spaces between cells) or active endocytosis, particularly in root hairs [63]. Previous studies have demonstrated that nanoplastics (<100 nm) can be taken up by plant roots, reaching vascular tissues in *Arabidopsis thaliana* [64]. Although the MPs used in this study are in the micrometer range, they may still adhere to or become embedded within root tissues under specific conditions.

The water uptake process may also facilitate MP–root interactions. MPs suspended in water can be transported toward root surfaces via capillary action, particularly under conditions of high transpiration rates. Once near the root zone, MPs may be physically retained within root hairs or adhered to due to localized charge variations influenced by pH gradients in the rhizosphere [65].

These mechanisms collectively suggest that aged and pristine MPs with adsorbed ATZ may interact with plant roots through a combination of hydrophobic effects, biochemical interactions, microbial colonization, and water flow dynamics. Further research is necessary to determine the extent of MP penetration into root tissues and whether these interactions affect a plant’s physiology, nutrient uptake, or toxicity responses.

These findings align with the mode of action of ATZ, a herbicide that inhibits photosynthesis by blocking electron transfer in Photosystem II, thereby reducing energy production and the essential organic compounds necessary for plant growth [6]. This disruption is intensified by interactions with MPs, particularly AMPs, which have a greater capacity to retain and release ATZ through direct contact with plant radicles. This dynamic highlights how MPs, both PMPs and AMPs, act as transport vectors, amplifying localized exposure and exacerbating phytotoxic impacts [66].

Phytotoxicity data also revealed significant differences in the germination indices and root lengths of *Lactuca sativa*, underscoring the deleterious effects caused by AMPs associated with ATZ [20,67].

## 4. Conclusions

This study investigated the sorption behavior of ATZ on PMP and AMP polyethylene MPs, analyzing its environmental and phytotoxic implications. The results demonstrated that AMPs exhibited a higher ATZ adsorption capacity than PMPs, which can be attributed to the structural modifications caused by photodegradation, such as the formation of oxygenated functional groups and increased surface roughness, as confirmed by FTIR and SEM analyses.

The phytotoxicity assays revealed that ATZ adsorbed onto MPs significantly inhibited the germination of *Lactuca sativa*. This effect was more pronounced for the AMPs compared to the PMPs or ATZ alone, indicating that the AMPs act as an enhanced vector for ATZ delivery to plant systems. Experimental observations suggest that the increased phytotoxicity stems from both the stronger physicochemical interactions between AMPs and ATZ, which enhance herbicide retention and transfer, and the direct interference of MPs in root physiology. Specifically, exposure to AMP-bound ATZ resulted in reduced root elongation and altered root morphology, likely due to oxidative stress and metabolic disruption caused by prolonged ATZ contact.

Furthermore, our findings demonstrate that MPs in the rhizosphere can physically interact with root surfaces through adhesion mechanisms influenced by root exudates, mucilage-mediated attachment, and capillary forces. This interaction may lead to mechanical stress, blockage of root hairs, and disruption of water and nutrient uptake [68]. Additionally, AMP-induced biofilm formation could alter the microbial communities critical for plant health, further compounding the toxic effects. These observations align with previous studies [69,70] suggesting that AMPs exhibit a higher affinity for plant roots due to surface oxidation and charge modifications, intensifying both root entrapment and pollutant uptake.

From a chemical perspective, the adsorption of ATZ onto MPs increases its environmental persistence and bioavailability in the root zone, thereby prolonging plant exposure to this herbicide. This extended exposure was experimentally linked to increased oxidative stress markers in plant tissues and hindered seedling development. These results indicate that MPs not only serve as passive carriers of ATZ but also actively modulate its environmental fate and phytotoxic effects. Given that AMPs adsorb more ATZ than PMPs, their presence in contaminated environments could exacerbate the long-term ecological risks associated with pesticide exposure, particularly in agricultural regions with intensive herbicide application.

Moreover, our results highlight that MPs significantly enhance the persistence of ATZ in aquatic environments, thereby increasing its bioavailability to aquatic organisms. This interaction may amplify the ecological risks posed by both pollutants, particularly in agricultural runoff areas where MPs and ATZ co-occur. Additionally, ATZ was observed to exert direct toxic effects on plants through root contact, raising concerns about its potential uptake in crops and subsequent impacts on plant health and productivity. The presence of MPs further modulates this process by altering ATZ mobility and bioavailability in soil and water matrices, emphasizing the need for further research on its implications for food safety.

Overall, these findings underscore the dual threat posed by MP-bound ATZ, as MP accumulation in the rhizosphere induces mechanical stress and disrupts root nutrient uptake, while ATZ release from MPs enhances phytotoxicity through direct root exposure.

Future research should focus on the mechanisms underlying MP–root interactions, particularly concerning MP penetration into root tissues and its effects on plant metabolism and nutrient absorption. Additionally, assessing the potential entry of MP-bound ATZ into food chains is crucial for understanding its broader ecological and human health implications.

Also, our results indicate that MPs significantly enhance the persistence of ATZ in aquatic environments, which could increase its bioavailability to aquatic organisms. This interaction may exacerbate the long-term ecological risks associated with both pollutants, particularly in agricultural runoff areas. Additionally, ATZ has been revealed to act directly on plants through root contact, which raises concerns about its uptake in crops and potential impacts on plant health and productivity. The presence of MPs may further influence this process by altering the availability and mobility of ATZ in soil and water matrices. Future studies should explore the potential for MP-bound ATZ to enter food chains, as well as mitigation strategies for reducing such contamination

These insights are critical for designing sustainable environmental management strategies aimed at reducing MP pollution and minimizing pesticide-associated risks in terrestrial and aquatic ecosystems.

## Figures and Tables

**Table 1:** Toxicity classification [40].

IGN (%) and IER (%)	Toxicity
0 to −0.25	Low
−0.25 to −0.5	Moderate
−0.5 ato−0.75	High
−0.75 ato −1.0	Very high

**Table 2:** Kinetic adsorption parameters of ATZ onto PMPs and AMPs under different aquatic matrices: DW and EW.

Matrix	Control	AV	Pseudo-First-Order			Pseudo-Second-Order		
			*q_e_*	*k*_1_	*R* ^2^	*q_e_*	*k*_2_	*R* ^2^
DW	ATZ	PMP	0.427	0.07	0.9773	0.440	0.37	0.9833
DW	ATZ	AMP	0.505	0.100	0.9680	0.522	0.42	0.9877
EW	ATZ	PMP	0.566	0.197	0.9985	0.572	1.75	0.9998
EW	ATZ	AMP	0.603	0.06	0.9758	0.623	0.18	0.9869

*q_e_* (mg g^−1^); *k*_1_ (h^−1^); *k*_2_ (g mg^−1^ h^−1^).

**Table 3:** Isothermal adsorption parameters of ATZ onto PMPs and AMPs under different aquatic matrices: DW and EW.

Matrix	Control	AV	Langmuir			Freundlich		
			*K_L_*	*q_m_*	*R* ^2^	*K_F_*	*n*	*Adj. R* ^2^
DW	ATZ	PMP	0.145	1.612	0.997	0.196	1.201	0.991
DW	ATZ	AMP	0.671	0.841	0.975	0.300	1.679	0.922
EW	ATZ	PMP	0.204	1.493	0.953	0.239	1.272	0.934
EW	ATZ	AMP	0.495	1.076	0.948	0.321	1.584	0.901

*K_L_* (L/mg); *q_m_* (mg/g); *K_F_* (L/g); *n* adimensional.

**Table 4:** Root length evolution.

DW	**Treatments**	**Average**	**Average Control**	**% Germinated**	**Germin. Control (%)**	**IER**	**IGN**	**Toxicity**
	ATZ	2.49	3.07	83	100	−0.19	−0.17	Low
	PMP	2.79		90		−0.09	−0.10	Low
	AMP	2.66		87		−0.13	−0.13	Low
	PMP+ATZ	1.82		73		−0.41	−0.27	Moderate T
	AMP+ATZ	1.68		67		−0.45	−0.33	Moderate
EW	**Treatments**	**Average**	**Average Control**	**% Germinated**	**Germi. Control (%)**	**IER**	**IGN**	**Toxicity**
	ATZ	2.72	3.07	87	100	−0.11	−0.13	Low
	PMP	2.75		93		−0.10	−0.07	Low
	AMP	2.65		93		−0.14	−0.07	Low
	PMP+ATZ	1.87		70		−0.39	−0.30	Moderate
	AMP+ATZ	1.86		70		−0.39	−0.30	Moderate

## Data Availability

Data will be available upon request to the authors.

## Abbreviations

The following abbreviations are used in this manuscript:

ATZ	Atrazine
MP	Microplastic
AMP	Aged microplastic
PMP	Pristine microplastic
DW	Distilled water
EW	Enriched water
CI	Carbonyl index
qt	Adsorption capacity
Co	Initial concentration
Ct	Final concentration
m	Mass of microplastic
PFO	Pseudo-first-order
PSO	Pseudo-second-order
ICR	Root Growth Index.
CRA	Root Growth in Sample.
CRC	Root Growth in the Control.
IG	Germination Index.
SGA	Number of Germinated Seeds in Sample.
SGC	Number of Germinated Seeds in Negative Control.
IER	Normalized Root Growth Percentage Index.
CMRCA	Average Radicle Length in Each Sample.
CMRCN	Average Radicle Length of Germinated Seeds in the Control.
IGN	Normalized Residual Germination Percentage Index
GERM	Germinated Seeds.
SEM	Scanning electron microscopy
FTIR	Fourier transform infrared spectroscopy
PE	Polystyrene
ZP	Zeta potential
